# Sex Differences in the Association of HOMA-IR Index and BDNF in Han Chinese Patients With Chronic Schizophrenia

**DOI:** 10.3389/fpsyt.2021.656230

**Published:** 2021-06-21

**Authors:** Yating Yang, Yulong Zhang, Juan Wang, Xiaoshuai Ning, Yelei Zhang, Tongtong Zhao, Yi Zhong, Zhiwei Liu, Lei Xia, Wenzheng Li, Xianhu Yao, Kai Zhang, Huanzhong Liu

**Affiliations:** ^1^Department of Psychiatry, Chaohu Hospital of Anhui Medical University, Chaohu, China; ^2^Anhui Psychiatric Center, Anhui Medical University, Chaohu, China; ^3^Chengdu Fourth People's Hospital, Chengdu, China; ^4^Hangzhou Seventh People's Hospital, Hangzhou, China; ^5^Fuyang Third People's Hospital, Fuyang, China; ^6^Hefei Fourth People's Hospital, Hefei, China; ^7^Ma'anshan Fourth People's Hospital, Ma'anshan, China

**Keywords:** HOMA-IR, BDNF, schizophrenia, sex differences, metabolism

## Abstract

**Background:** Previous research has indicated that there are significant sex differences in serum BDNF levels and metabolic indicators in patients with schizophrenia. Studies have found that BDNF is involved in blood sugar regulation. Homeostasis model assessment of insulin resistance (HOMA-IR) is currently a sensitive indicator for measuring insulin resistance. Our study aims to explore the sex differences in the relationship between serum BDNF levels and HOMA-IR in patients with chronic schizophrenia (CS).

**Methods:** A total of 332 patients with CS were enrolled in this study. General information of all participants was collected. Haematological indicators were collected, and the Positive and Negative Syndrome Scale (PANSS) was used to evaluate psychiatric symptoms. Sex differences in serum BDNF levels, HOMA-IR index and other metabolic indexes were investigated. Then, linear regression analysis was used to analyse the relationship between the HOMA-IR index and BDNF levels in male and female patients.

**Results:** The HOMA-IR index of female patients was significantly higher than that of males, but there was no significant difference in serum BDNF levels between male patients and female patients. There was a positive correlation between BDNF level and HOMA-IR index, and this relationship only existed in female patients.

**Conclusion:** The results show that there are significant sex differences in HOMA-IR in patients with CS. In addition, only in female patients was there a positive correlation between the HOMA-IR index and BDNF level, which suggests that sex factors should be taken into account in evaluating the relationship between BDNF and blood glucose in patients with CS.

## Introduction

Schizophrenia has a high mortality and disability rate (1, 2). The prevalence rate of schizophrenia in China is approximately 0.7% (3). Studies have shown that sex differences are widespread in patients with schizophrenia. For example, there are significant sex differences not only in the age of onset, the efficacy of drug therapy and the severity of symptoms but also in neurocognitive functioning (4–6). At the same time, our research also shows that there are significant sex differences in metabolism in patients with schizophrenia (7–9). For example, the prevalence of hyperhomocysteinemia in male patients with CS is significantly higher than that in female patients (10). In addition, our previous studies have found that female patients with CS are more likely to be obese, whereas male patients are more likely to be underweight (11).

A study of 272,248 patients with schizophrenia found that approximately 3/4 of patients died of natural causes (12). However, the most common cause of premature natural death was cardiovascular disease, and people with schizophrenia had a higher risk of cardiovascular disease than the general population (13, 14). Insulin resistance (IR) can cause a series of metabolic abnormalities that lead to metabolic syndrome and type 2 diabetes. At present, the homeostasis model assessment of insulin resistance (HOMA-IR) is widely used by clinicians to evaluate IR.

On the one hand, a recent meta-analysis of 65 studies (516,325 patients) found that IR can be used as a predictor of cardiovascular disease (15). A prospective study of 2,983 subjects showed that people with IR had 1.4 times more cardiovascular events than those without IR, and the risk of diabetes was 1.5 times higher than that of those without IR (16). Moreover, another study found that effective prevention of IR can prevent 42% of myocardial infarctions (17). In summary, IR is not only a risk factor for diabetes and cardiovascular disease but also an important predictor of cardiovascular events. Previous studies have found that the prevalence of insulin resistance is also significantly higher in first-episode schizophrenia patients (18). On the other hand, epidemiological studies have shown that IR is related to sex. Women whose mothers had diabetes were more likely to develop diabetes than women whose fathers had diabetes (19). This begs the question of whether this sex difference in glucose metabolism also exists in patients with schizophrenia.

Brain-derived neurotrophic factor (BDNF) is a kind of energy balance regulator that also plays an important role in the central regulation of food intake and body weight (20, 21). Moreover, BDNF is an important member of the neurotropic factor family, which is widely distributed in the central nervous system (22). BDNF also plays a critical role in the neurotransmission of dopaminergic and serotonergic neurons, especially by regulating the differentiation of dopaminergic neurons, thus affecting the release of dopamine and serotonin (23, 24). Furthermore, additional studies have reported that changes in peripheral BDNF levels may be related to the pathophysiology of patients with schizophrenia (25, 26). For example, BDNF is associated with positive symptoms (27) and negative symptoms (28). Previous studies have shown that BDNF can cross the blood-brain barrier (29, 30). In addition, animal experiments have shown that blood BDNF levels are positively correlated with brain BDNF levels (31). Interestingly, many studies have found significant sex differences in BDNF levels in patients with schizophrenia (20, 32). Moreover, there were significant sex differences in the correlation between BDNF and cognition in patients with schizophrenia (33).

Previous studies have found that BDNF can reduce food intake and blood sugar in obese diabetic animal models (34, 35), and BDNF has a hypoglycaemic effect independent of appetite changes (36). In addition, in an experimental study, changes in the BDNF signalling pathway were associated with insulin resistance and hyperglycaemia (36). In rodents, changes in the BDNF signalling pathway lead to hyperglycaemia (37). The BDNF effect may play a role by increasing the insulin sensitivity of peripheral tissue (38). The above studies show that there is a close relationship between BDNF and glucose metabolism. However, the relationship between BDNF level and HOMA-IR index in patients with CS is not clear; therefore, the purpose of our study was to use the HOMA-IR index to express IR and to investigate the relationship between the HOMA-IR index and plasma BDNF level in patients with CS. We also analysed whether there were sex differences in the relationship between the HOMA-IR index and plasma BDNF levels and explored the influencing factors of HOMA-IR in patients of different sexes with CS.

## Method

### Subject

From May 2018 to December 2018, 332 patients (136 women and 196 men) with CS were selected from three hospitals in the Anhui Province, China (Chaohu Hospital affiliated with Anhui Medical University, the Fourth People's Hospital of Maanshan, and the Fourth People's Hospital of Hefei). Our admission criteria were as follows: (1) patients 18–75 years old; (2) patients meeting the diagnosis of schizophrenia based on the Diagnostic and Statistical Manual of Mental Disorders (DSM-V); and (3) patients with an illness duration of more than 5 years (the course of disease ≥ 5 years as chronic patients) (39). The exclusion criteria were as follows: (1) pregnant or lactating women; (2) patients meeting the DSM-V criteria for other serious mental disorders, except schizophrenia, such as depressive disorder and obsessive-compulsive disorder; (3) those with severe somatic or infectious diseases; and (4) those who were taking non-steroidal anti-inflammatory drugs, corticosteroids or other immunomodulatory agents. This study was approved by the Ethics Committee (Chaohu Hospital of Anhui Medical University; argument number: 201805-KYXM-03). This study was registered with the China Clinical Trial Registration Center (chiCTR 1800017044).

### Demographic Characteristics

Through interviews, four researchers collected the general information of the patients (e.g., sex, age, years of education, illness duration, smoking). The height and weight of the participants were measured when they wore light clothes and went barefoot. Body mass index (BMI) was calculated by weight (kg)/[height (m) ^2^]. Following the criteria of the Working Group on Obesity in China (WGOC), individuals were categorised by BMI as follows: obesity, BMI ≥ 28 kg/m^2^. As a recognised sensitive index of insulin sensitivity and abnormal glucose regulation, the HOMA-IR index is calculated from the levels of venous blood glucose and insulin: HOMA-IR = [fasting insulin (μIU/ml) × fasting blood-glucose (mmol/L)]/22.5 (40).

### Scale Evaluation

The Positive and Negative Syndrome Scale (PANSS) was used to evaluate the severity of schizophrenia (41). The scale has a total of 30 items, with the highest score of 7 and the lowest score of 1. We used a three-factor model, which was divided into general psychopathology (7 items), positive symptoms (16 items) and negative symptoms (7 items), and the symptom severity was positively correlated with the score. Four psychiatrists received consistency training, and the internal consistency coefficient was >0.8. In this study, chlorpromazine equivalent was used to express the dose of antipsychotic drugs in patients with CS. Regarding the types of antipsychotic drugs, they were divided into typical antipsychotic drugs (perphenazine and sulpiride), atypical antipsychotic drugs (clozapine, olanzapine, quetiapine, aripiprazole, ziprasidone, aminosulfonyl) and combination drugs (taking two or more antipsychotic drugs).

### Biochemical Assays

Whole fasting venous blood samples of all participants were collected from 7:00 to 8:00 in the morning. After collection, the samples were numbered, centrifuged (rpm = 3,600, 10 min) and separated into serum. The samples were stored at −80°C, and the indexes were tested within 30 days. Sandwich enzyme-linked immunosorbent assay (ELISA) (human ELISA kit; CUSABIO Science, Wuhan, China) was used to determine the level of BDNF in 332 patients with schizophrenia. The results are expressed as ng/ml. BDNF was <8%, whereas the interassay coefficients of variation were <10%. The measurement methods of other biochemical indexes were as follows: total cholesterol (TC) was measured by the CHOD–POD method, and triglycerides (TGs) were detected by the GPO-PAP method (Beijing Liedman Biochemical Company, Beijing, China). High-density lipoprotein (HDL) and low-density lipoprotein (LDL) were measured by the terminal method. Determination of fasting blood glucose (FBG) was performed by the oxidase method (Zhejiang Meikang Biotechnology Company). Plasma insulin was determined by electrochemiluminescence (Mannheim Roche Diagnostics Company).

### Statistical Analysis

First, SPSS 23 was employed to conduct the statistical analysis of the study. Continuous variables are expressed as the mean ± SD, and categorical variables are expressed as percentages. Second, we compared the general data (age, course of disease, years of education, and marital status) and blood parameters (FBG, TG, TC, HDL, LDL, BDNF, and HOMA-IR index) between male and female patients. The chi-square test was used for classified variables, and a *t*-test was used for continuous variables in accordance with a positive etheric distribution; otherwise, the Mann–Whitney *U*-test was used. Third, sex differences in the HOMA-IR in all patients were compared, and covariance analysis was carried out with the HOMA-IR index as the dependent variable and age, PANSS total score, negative scale score, positive scale score, general pathology score, TC, TG, HDL, LDL, BDNF, illness duration, antipsychotic dose, and antipsychotic class as covariates. The same method was used to analyse the sex differences in BDNF in all patients and covariance. Finally, for the linear regression analysis of female patients, we input general demographic data and clinical variables (e.g., age, PANSS total score, negative scale score, positive scale score, general pathology score, TC, TG, HDL, LDL, BDNF, illness duration, antipsychotic dose, antipsychotic class) to predict the level of the HOMA-IR index. The same method was used to analyse the influencing factors of the HOMA-IR index in male patients. A two-tailed *P* < 0.05 indicated significance.

## Results

### Comparison Between Male and Female Patients

The differences in sociodemographic data and clinical characteristics between male and female patients are shown in Table 1. A total of 332 patients with CS participated in this study (136 women and 196 men). The average age of female patients and male patients was 45.88 ± 11.62 and 44.59 ± 11.89, respectively. The average illness duration of female patients was 19.1 ± 10.78, whereas the average total course of male patients was 19.01 ± 10.24. There was no significant difference between the two groups. Compared with female patients, male patients were more likely to be smokers, but the TC, HDL, and LDL in male patients were lower than those in female patients. In addition, compared with male patients, female patients had relatively mild psychiatric symptoms, such as lower PANSS total scores, negative symptom scores and general psychopathology scores. In addition, all *P* values <0.05.

**Table 1:** Sociodemographic and clinical characteristics of male and female patients with CS (*n* = 332).

**Variable**	**Female (*n* = 136)**	**Male (*n* = 196)**	***t/Z/**χ^2^*****	***P***
Age (years)	45.88 ± 11.62	44.59 ± 11.87	−1.192	0.233
IR (%)	59 (44.7%)	61 (32.1%)	5.282	**0.022**
Age of onset	26.53 ± 8.644	25.66 ± 7.926	−0.687	0.492
Illness duration	19.1 ± 10.78	19.01 ± 10.24	−0.033	0.974
Education (years)	8.29 ± 3.91	8.02 ± 3.83	−0.761	0.446
Smoking (%)	1 (0.7%)	96 (49.7%)	92.156	** <0.001**
Obesity (%)	30 (22.1%)	23 (11.7%)	6.379	**0.012**
TC (mmol/l)	4.95 ± 1.48	4.62 ± 1.35	−2.114	**0.035**
TG (mmol/l)	2.37 ± 1.67	2.15 ± 1.39	−1.563	0.118
HDL (mmol/l)	1.11 ± 0.26	1.01 ± 0.26	−3.69	** <0.001**
LDL (mmol/l)	2.46 ± 0.59	2.35 ± 0.65	−2.117	**0.034**
FBG (mmol/l)	5.44 ± 1.52	5.28 ± 1.21	−0.249	0.803
HOMA-IR	2.67 ± 2.1	1.93 ± 1.36	−3.321	**0.001**
BDNF	2.17 ± 2.36	2.12 ± 3.02	−1.965	**0.049**
Antipsychotic dose	500.86 ± 283.15	424.06 ± 244.02	−2.487	**0.013**
Antipsychotic drug (%)			2.193	0.334
Typical	3 (42.9%)	4 (57.1%)		
Atypical	50 (36.2%)	88 (63.8%)		
Combination	83 (44.4%)	104 (55.6%)		
PANSS total	72.83 ± 23.32	81.36 ± 24.19	−3.082	**0.002**
Positive subscale	16.99 ± 6.84	18.66 ± 7.76	−1.79	0.073
Negative subscale	19.77 ± 7.26	22.77 ± 7.62	−2.98	**0.003**
General psychopathology subscale	36.12 ± 12.10	39.94 ± 12.92	−2.64	**0.008**

*Bold value means P <0.05*.

### Sex Differences Between the HOMA-IR Index and BDNF

**Figure 1:**
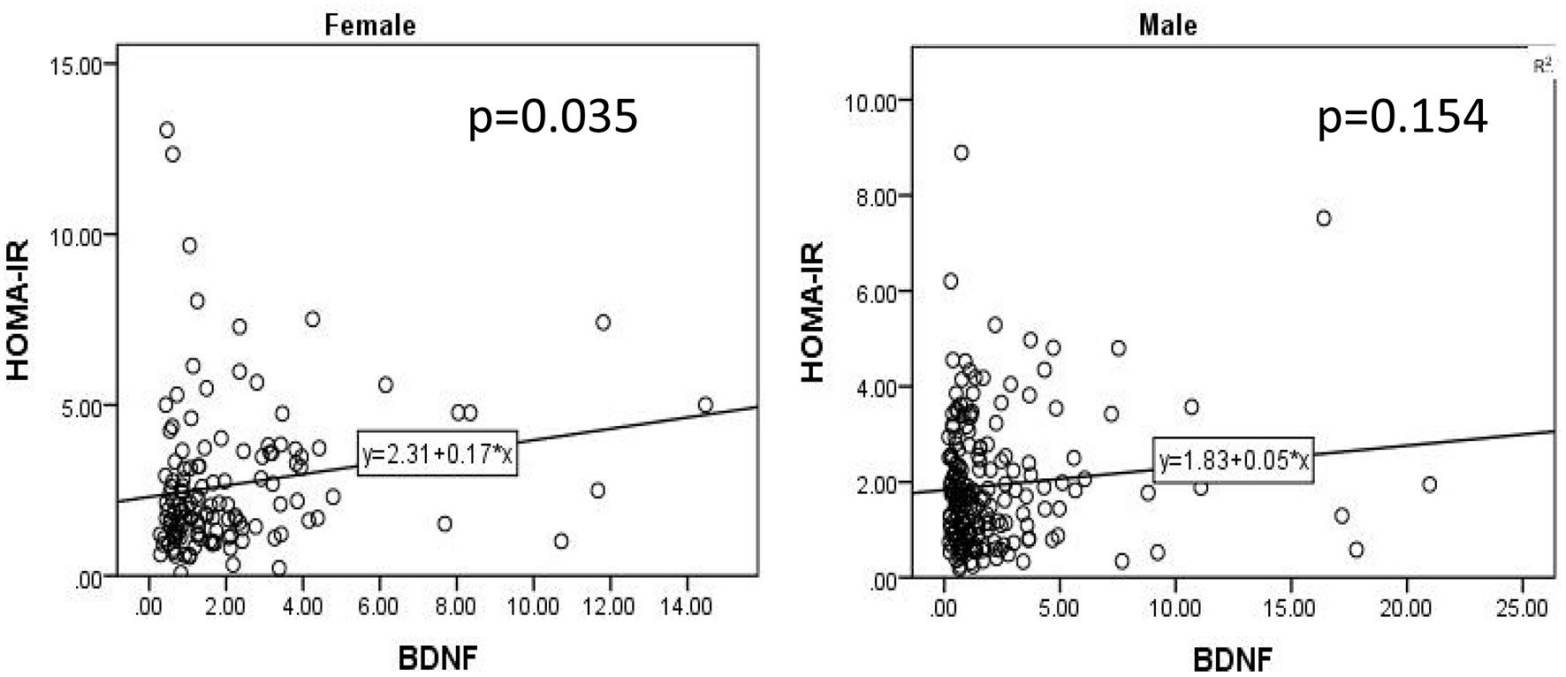
There was a significant positive correlation between HOMA-IR index and BDNF level in female patients with chronic schizophrenia (*r* = 0.17, *P* = 0.035), while there was no significant correlation between HOMA-IR index and BDNF level in male patients (*r* = 0.05, *P* = 0.154).

As shown in Table 1, female patients had a relatively higher HOMA-IR index and BDNF than male patients. The covariance analysis with the HOMA-IR index as the dependent variable showed that the HOMA-IR index of female patients was still higher than that of male patients (*F* = 9.245, *P* = 0.003). Except for sex, BMI (*F* = 17.831, *P* < 0.001), age of onset (*F* = 6.905, *P* = 0.009) and course of disease (*F* = 7.548, *P* = 0.006) were all influencing factors of the HOMA-IR index. Using the same method, to further compare sex differences in BDNF levels, covariance analysis was carried out with BDNF levels as the dependent variable and other variables as covariates. The results showed that there was no significant difference in BDNF levels between male and female patients (*F* < 0.001, *P* = 0.985). The results of the covariance analysis showed that BMI was the factor affecting the level of BDNF (*F* = 4.005, *P* = 0.046). There was a significant positive correlation between HOMA-IR index and BDNF level in female patients with chronic schizophrenia (*r* = 0.17, *P* = 0.035), while there was no significant correlation between HOMA-IR index and BDNF level in male patients (*r* = 0.05, *P* = 0.154) (Figure 1).

### Sex Differences in the Relationship Between BDNF and the HOMA-IR Index

For the linear regression analysis of female patients, we input general demographic data and clinical variables (PANSS total score, negative scale score, positive scale score, general pathology score, TC, TG, HDL, LDL, BDNF) to predict the level of the HOMA-IR index. BMI, HDL and BDNF were the only three variables that made significant contributions to the prediction of the HOMA-IR level, *F*_(2, 11)_ = 2.588. The *R*^2^ of the overall model was 22.2%, and the adjusted *R*^2^ was 13.6%, which is a moderate effect according to Cohen et al. (42). The specific results are shown in Table 2.

**Table 2:** Linear regression analysis of serum BDNF and HOMA-IR index in female patients with CS.

**Female**	**β**	**SE**	***t***	***P***
HDL	−2.309	0.810	−2.309	**0.023**
BDNF	0.203	0.081	2.253	**0.026**
BMI	0.242	0.050	2.732	**0.007**
Age	−0.146	0.022	−1.219	0.225
Illness duration	0.025	0.024	0.205	0.838
TC	−0.104	0.158	0.952	0.343
TG	0.009	0.122	0.091	0.927
LDL	0.109	0.368	1.076	0.284
Positive subscale	−0.193	0.351	−0.170	0.865
Negative subscale	−0.325	0.344	−0.276	0.783
General psychopathology subscale	−0.529	0.344	−0.267	0.790
PANSS total	0.834	0.345	0.218	0.828
Antipsychotic dose	−0.087	0.001	−0.970	0.334
*R*^2^	0.136			

*Bold value means P <0.05*.

Similar linear regression analysis was performed on male patients. We entered the clinical variables of general demographic data (PANSS total score, negative scale score, positive scale score, general pathology score, TC, TG, HDL, LDL, BDNF, smoking status) to predict the HOMA-IR level. Only BMI, TG, and LDL played important roles in the prediction of the HOMA-IR level, *F*_(2, 15)_ = 8.792. The *R*^2^ of the overall model was 37.5%, and the adjusted *R*^2^ was 33.2%. It was a moderate effect. The specific results are shown in Table 3.

**Table 3:** Linear regression analysis of serum BDNF and HOMA-IR index in male patients with CS.

**Male**	**β**	**SE**	***t***	***p***
Age	−0.136	0.010	−1.495	0.137
Illness duration	0.013	0.012	0.139	0.890
TC	−0.109	0.092	1.181	0.239
TG	0.170	0.066	2.484	**0.014**
HDL	0.095	0.371	1.311	**0.192**
LDL	0.170	0.80	0.946	0.053
Positive subscale	0.032	0.015	0.357	0.722
Negative subscale	0.070	0.016	0.770	0.442
General psychopathology subscale	−0.209	0.012	−1.799	0.074
BDNF	0.034	0.028	−0.533	0.595
BMI	0.489	0.025	6.968	** <0.001**
Antipsychotic dose	−0.046	0.001	−0.706	0.481
*R*^2^	0.332			

*Bold value means P <0.05*.

## Discussion

This study found that (1) there were significant sex differences in the HOMA-IR index in patients with CS, and the level of HOMA-IR in female patients was significantly higher than that in male patients. (2) There were significant sex differences in the correlation between BDNF and the HOMA-IR index, and this correlation only existed in female patients. (3) The above findings also suggest that we should pay attention to sex factors when evaluating the metabolic indexes of patients with CS in future studies.

Our study found that the average HOMA-IR index of patients with CS was 2.23 ± 1.75 ng/ml. Furthermore, some scholars investigated 44 healthy controls and 70 patients with first-episode untreated schizophrenia and found that the HOMA-IR of healthy people and patients with schizophrenia were 1.28 ± 0.52 and 1.83 ± 1.16 ng/ml, respectively (39). The HOMA-IR index is an effective and extensive index to evaluate IR according to fasting blood glucose status in epidemiological studies, and it is widely used in clinical practice (43–45). However, there is currently no specific scope for the HOMA-IR index to assess IR. Lin et al. found that if a HOMA-IR index >1.7 was defined as IR, the prevalence of IR in patients with schizophrenia was 37.82% (46). Recently, a meta-analysis (including 731 patients with schizophrenia and 614 individuals from the general population) showed that patients with first-episode schizophrenia had significantly higher IR than the general population and an increased risk of diabetes (47). In summary, patients with CS have higher HOMA-IR levels and are more likely to develop IR than the general population. This may be related to the use of second-generation antipsychotics (48) and the unhealthy lifestyle of patients with schizophrenia, such as sedentary lifestyle (49), poor diet (50), lack of exercise and other unhealthy habits (51). Antipsychotic drugs, as a special factor in patients with schizophrenia, significantly increase the incidence of metabolic diseases in patients with schizophrenia. For example, a meta-analysis compared the risk of diabetes in patients with schizophrenia after taking different antipsychotics, confirming that patients treated with second-generation antipsychotics had an increased incidence of diabetes (52). Olanzapine and clozapine are more likely to cause metabolic side effects than other antipsychotics (53, 54). In addition, some studies have found that the IR of first-episode schizophrenia patients was significantly higher than that of the general population (55). This shows that there is a complex relationship between IR, abnormal glucose metabolism and schizophrenia that needs more research to explore. However, our study did not control the types of antipsychotics in patients with CS, so we cannot explain the effects of antipsychotics on the HOMA-IR index and BDNF levels in patients with CS.

Additionally, our results show that there are significant sex differences in the HOMA-IR index of patients with CS. The HOMA-IR index of female patients was significantly higher than that of male patients, and through the analysis of covariance, this difference remained. This difference is reported for the first time and may be due to a normal physiological range of sex hormone levels that determine insulin sensitivity. An increase in relative androgen levels in women and a decrease in testosterone levels in adult men can cause IR (56). In addition, some studies have shown that women's insulin sensitivity decreases with the advent of menopause, and insulin sensitivity is improved through oestrogen replacement therapy, which also suggests that oestrogen may play an important role in female insulin sensitivity (57, 58). However, inconsistent with our results, the results of Lin et al. showed that there were no significant sex difference in the HOMA-IR index in patients with CS (46). We suspect that this may be because the average age of female patients with CS was 45.88 ± 11.62. They are in the perimenopausal period and lose the protective effect of oestrogen (59), so the HOMA-IR index of female patients is significantly higher than that of male patients.

Previous studies have found that there are significant gender differences in BDNF levels in patients with CS (60). At the same time, the results of Lin et al. showed that there were significant sex differences in neuronal expression and functional regulation of BDNF in animal models (61). However, previous studies have shown that this sex difference exists not only in patients with schizophrenia but also in patients with depression (62) and autistic spectrum disorders (63). Similar to the above results, Xiu et al.'s results showed that there were no sex differences in BDNF levels in the general population, but there was such a difference in patients with schizophrenia (32). However, after controlling for other variables through an analysis of covariance, our study found that there was no significant difference in BDNF levels between male and female patients. Consistent with the results of our study, Nurjono et al. (64) found that there was no significant sex difference in BDNF levels in patients with schizophrenia, which also indicates that the sex differences in BDNF in patients with schizophrenia are not consistent with the results of different scholars and that BDNF levels are affected by many factors *in vivo*, which requires an increasing number of accurate studies to explore.

It is well-known that antipsychotics have obvious regulatory effects on BDNF levels. For example, an animal experiment showed that typical and atypical antipsychotics had different regulatory effects on BDNF mRNA expression in the hippocampus (65) and neocortex of rats. Olanzapine or quetiapine seem to be beneficial to regulate the expression of BDNF in atypical antipsychotics (66, 67). Previous studies have found that serum BDNF levels in patients with CS who took clozapine or typical antipsychotics were higher than those who took risperidone (68). Previous studies have shown that BDNF levels in patients with CS who took clozapine were significantly higher than those who took typical antipsychotics (69). However, a survey showed that there was no change in serum BDNF in patients with schizophrenia after taking risperidone (70). The inconsistency of the above results suggests that the effect of antipsychotics on the level of BDNF in patients with schizophrenia is worthy of further exploration.

For the first time, we found that there were significant sex differences in the association between HOM-IR and BDNF levels. In our study, we found that there was a positive correlation between HOMA-IR and BDNF levels only in female patients. We further conducted multiple regression analysis on male and female patients and found that HDL and BDNF levels could predict the HOMA-IR index in female patients. However, in male patients, we found that TG, obesity and LDL levels could predict HOMA-IR index levels. This shows that BDNF is an important influencing factor of the HOMA-IR index in female patients, rather than male patients. Boyuk et al. found that in patients with type 2 diabetes, the higher the blood sugar, the higher the serum BDNF level (71). In recent years, some scholars have found that increasing the level of peripheral BDNF had a hypoglycaemic effect on obese and hyperglycaemic mice, but it had no effect on healthy mice (34, 36, 72). Combined with animal experiments, we speculate that when blood sugar rises, the increase in BDNF has a protective effect on the body. In addition, Yang et al. showed that there are obvious sex differences in the relationship between BDNF and BMI (20). This also reminds us to consider sex factors when discussing the relationship between BDNF and metabolism. However, the specific mechanism of sex differences needs more precise experiments to verify.

Patients with schizophrenia are more likely to develop IR than the general population, but the specific mechanism of IR in patients with CS is not clear. This study found that there were significant sex differences in the HOMA-IR index in patients with CS, and there were significant sex differences in the relationship between BDNF and CS. This study provides a reference for follow-up studies and reminds us that we need to pay attention to sex differences in CS with IR in clinical work.

However, our study has some limitations. First, it is a cross-sectional study, and we cannot obtain a specific causal relationship. Second, our subjects included only the Chinese Han population, so the results are difficult to apply to other ethnic groups. Third, our study covers only patients with CS, and these patients are hospitalised for a long time, which biases the results. More accurate studies are needed to validate our research. Fourth, the number of female patients included in our study was significantly smaller than that of male patients, which may have affected the results of the study. Fifth, this study did not evaluate whether female patients are postmenopausal or measure their hormone levels. Sixth, this paper did not investigate whether patients had comorbidities with other specific diseases, such as hypercholesterolemia, hypertriglyceridemia and other somatic diseases, as these diseases have a significant impact on the HOMA-IR index of patients.

## Conclusion

In conclusion, our research results show that there are significant gender differences in the relationship between HOMA-IR and BDNF in patients with CS. When evaluating the relationship between BDNF levels and metabolic indicators in patients with CS, gender factors need to be considered.

## Data Availability Statement

The raw data supporting the conclusions of this article will be made available by the authors, without undue reservation.

## Ethics Statement

The studies involving human participants were reviewed and approved by Human Research and Ethics Committee of Chaohu Hospital of Anhui Medical University (201805-kyxm-03). The patients/participants provided their written informed consent to participate in this study.

## Author Contributions

YY, YZ, JW, KZ, and HL collected and statistically analyzed the data, and wrote the first draft, which was revised and approved by all authors.

## Conflict of Interest

The authors declare that the research was conducted in the absence of any commercial or financial relationships that could be construed as a potential conflict of interest.
